# Psychological Quarterly Retrospect

**Published:** 1859-04-01

**Authors:** 


### *Psychological Quarterly Retrospect.*

At no time may the psychical character of a period be so well observed as when events occur the influence of which upon the thoughts and feelings both of individuals and the mass had already been previously well ascertained. In the political vocabulary there is a word which, from its potent character, we rarely write—that is, in its political sense—without feeling an inclination to put it in black letter; for the most powerful charm ever wielded by Circe, the most effective rune of a Scandinavian skald, and the blackest incantation of witch or wizard never excited so great a turmoil in the nether world as that solitary word has been accustomed to excite among the people of this country. Once let Reform be uttered by an experienced politician, and as if the nation had eaten of the insane root that takes the reason prisoner, order and quietude have been commonly driven to the winds; and too often the very dregs of popular disturbance have been tossed up to the surface in the commotion. But a change has come over the spirit of the people, and now, although for several weeks the charm has been iterated and reiterated, the surface of society has been barely ruffled by it. Whence comes this strange and gratifying change? Has the word lost its power, or have the friends of peace and quietude discovered a spell by which the evil spirit of agitation may be allayed—a spell powerful as that by which the sorcerer was accustomed to remit the foul fiend to his darksome region, *per ipsum, et cum ipso, et in ipso*. The *Times* (March 11), says that—

> "At present there is no desire for organic change. Revolutions across the Channel are said to be made by the belly, and in this country political discontent is always found to follow the stagnation of trade and the distress of the working people. Emigration and the progress of industry have removed the misery which caused former agitations, and in no quarter of the country can people be found to believe seriously that they are oppressed by the members for small boroughs, or that the aristocracy, as Mr. Bright gravely assures us, are nourished on our 70,000,000*l.* of taxation. . . . . But it does not follow that this will always be the temper of the people. A commercial crisis, a bad harvest, an ill-conducted war, may rouse the spirit of 1832 and 1846."

In truth, suffering, or ignorance, or both, lie at the root of all excessive popular commotions. To the diminution of ignorance we may fairly attribute a portion of that quietude which at present reigns in spite of the important political changes in progress, and we may ask if we are not already reaping some of the harvest which has arisen from the efforts that have been made on all hands for the amelioration of the social condition and for the intellectual culture of the mass of the people, and if certain items of national expenditure (called extravagances by a few popular orators) of the Government, have not in a great measure nipped in the bud the disposition for popular commotion. On the said expenditure, the *Times* has the following suggestive and truthful remarks :—

"We say that our public charges are usually incurred for good purposes, and less frequently at the suggestion of the Government than of the people themselves. The tendency to increase, which has lately pervaded all departments, has been due in a great degree to the agitation of benevolent and patriotic men. It has arisen from the solicitude extended to all classes of the people—to paupers, to criminals, to lunatics, to children; from the patronage bestowed upon art and science; from the impulse given to education; and from the general expansion of the machine of government which the progress of the nation rendered inevitable. It has been commonly said of late times that the duty of Ministers now consists in resisting expenditure rather than defending it, and that it is from the nation at large that the cry rises for better service, though better service brings larger costs."

An old proverb saith, "a full belly neither fights nor flies well;" and this is the secret of the indisposition which exists to popular commotions during periods of prosperity. It needs no dissertation to show how bound up the psychical state of the individual or of the mass is with the welfare of the digestive organs. The mind truly reflects the impoverishment or well-being of the stomach and of the system at large; it is irritable, irrational, uncontrollable, if the body be ill-fed; easy, careless, and indifferent, if well-fed. We cannot control fluctuations in trade, but we can tutor minds to a better knowledge than the conception that political changes are a panacea for social suffering of every class and grade; we can teach that there is a higher degree of reform than political reform; but it is well to remember that it is during periods of prosperity alone that this knowledge can be taught, and that efforts can be made for the intellectual and moral well-being of the nation. When suffering occurs, a better check than the law to the excesses of popular commotion is the restraint of a previous sound Christian education.

An article in the *Daily Telegraph* (February 4), on the horrible case of murder, arson, and suicide which occurred in Manchester on the 1st of February, contains an interesting outline of the psychical differences of the present period as compared with previous periods in this and the preceding century, so far as these differences may be illustrated by the records of murder :—

"The murders of the nineteenth century present a singular contrast to those of the eighteenth; and those of the third quarter are unlike those of the first and second. A hundred years ago existed the hags who cut the throats of children before stripping them of their clothes—the ogres who lurked in tumble-down houses near Drury-lane, to mutilate and burn the bodies of victims suffocated with pitch-plasters—the vampires who, prodigally paid by high-born ruffians from Westminster, made away with women and infants, and thus obliterated the evidence of still more infernal crimes. The highwayman shot the wayfarer through the brains; the masked burglar seldom broke open a desk before he clove the sleeper's skull; guilt was scarcely less ferocious than the law that punished it; but with a change in the penal statutes of the realm came an amelioration, even in the turpitude of the criminal classes. When Black Monday was no longer a hangman's revel at Execution Dock; when Tyburn ceased its exhibitions of brutality and death; when Edgeware-road saw the last of its immemorial gibbets disappear; the Dick Turpins of the road, and the Jack Sheppards of the town, the Burkes and Yarrows of Charing-cross, laid aside, for the most part, their bloody weapons, and made an effort, at least, to be burglars and footpads without being assassins. Some Thurtells and Rushes, no doubt, haunted the neighbourhood of wayside inns, and pistolled the farmer as he carried home the produce of a market day; but human life was never held so cheap after the capital penalty for secondary offences had been revoked. When to commit larceny was to risk the gallows, what was to check the desperate culprit when a blow or a stab might silence a dangerous witness? Our jurisprudence was improved, and crime, so to speak, reformed itself. It is true, of course, that within the actual century tragedies have taken place unsurpassed even in the blackest ages of Italian vengeance, or—which was worse—in the trap-door and dark-lantern epoch of London unlighted and unguarded. Not very many years have elapsed since Greenacre's ghastly baskets and packages were discovered, or since Daniel Good hid a woman's body, partly in a manger, and partly in the boot of a carriage. There was the grave dug by the Mannings under a floor; there was the Dublin slaughter of a cashier at his desk; there was the Rugeley Borgia, hideously prominent, the very type of a murderer; but atrocities of this description have become fewer. We hear of stabbings and poisonings, of strangulation and drowning; these, however, are, in most instances, the acts of passion, of jealousy, of hate, of exasperated egotism. Seldom, in our time, is man or woman slain in order to be robbed. And yet, to the elder generations, it seems not long since the West-country soldier saw a crippled girl receive eighteen pence in charity, tracked her into a forest, broke her neck, spent the money at a neighbouring tavern, was caught before his paroxysm of intoxication had been subdued, and hanged in chains on the very scene of his crime.

"These things are gradually passing away. When Kirwan stifled his wife, when Tawell mixed the dose of prussic acid, when Dove mimicked Palmer, when Bousefield, Corrigan, and Davis gave themselves up, in a moment of frenzy, to the gallows, it was not in the same spirit as that in which Courvoisier slew Lord William Russell. A deliberate murder, committed for the sake of a watch or a purse, is now scarcely ever heard of. The race to which Hocker belonged is dwindling out of sight. It re-appears, perhaps, in the form of Marley; but the characteristic crime of the day is that of assassination, committed in a state of madness, and followed, if not by suicide, at all events by a few days of unreasoning concealment and an apathetic surrender to the police. In this category stands Black, the Irish murderer, now awaiting justice; and some jurists are perplexed to decide between the policy of treating such as crimes of the highest degree, or as aberrations appealing to a wise and philosophical mercy. In the case of the imbecile murderer Dove, certain phrenological speculators endeavoured to screen him by declaring that he had been born with an imperfect mental, moral, and craniological constitution—to quote the slang of their pretentious science; but upon this assumption every flat-headed, large-eared, heavy-jowled, and monstrous villain in existence might plead for himself the misfortune of a defective cranium. We must apply other tests, or determine to establish vast prisons for the perpetual incarceration of murderers, who might then be subjected to the process recommended by an eminent social writer, and be compelled 'to work out their own biographies,' for the benefit of those abstract Cuviers and transcendental Owens who treat human nature as a symmetrical mechanism of motives, and pretend to infer, from a man's love of red as a colour, what crime he would be likely to commit, supposing any particular concatenation of circumstances.

"The Manchester tragedy, almost unique in its horror, belongs to the class we have described as characteristic of the present century. The crime was one of fury. William Robinson, landlord of the Cross Keys beer-house, in Albert-street, must have been a man of thoroughly sepulchral disposition. He was not a thief, or a sensualist, or accustomed to the commission of personal outrages; but he was a grim fellow, a fit bearer of the skull and crossbones. Nothing went wrong in his business, yet he would hire himself out as a mute or stagger under a coffin to a churchyard, more, it would seem, as a matter of amusement than of pay. He had formerly been a bailiff, but could never rest satisfied in any one place or capacity; however, after attending a funeral, it was his general custom to intoxicate himself at a tavern.

"After one of these ceremonies, with its dismal supplement of drink, Robinson went home on Tuesday evening last. In the same building, under the beer-shop, lived a woman named Mary Saxon, whose lot it is, while the dinner-controversy wages in the higher spheres, to sleep in a cellar. At the hour of three, while lying in bed, this woman heard a fall overhead; then she saw blood oozing through the floor. Immediately it occurred to her that Robinson was killing his wife; but there were two daughters expected home in an hour, and Mary Saxon most strangely forbore to give an alarm. She even saw the man walking up and down in front of his shop, with tears in his eyes, bare-headed, and evidently bewildered. Still the woman held her peace; three hours elapsed; the murderer shut himself in the house; the murdered creature lay on the stones in the kitchen; and at length, when the girls returned from their work at the factory, the dreary den was broken open.* It was found full of smoke and fire; flames were leaping from the gas-pipes, burning timbers were carrying the conflagration from room to room; Robinson's wife lay dead, the blood welling from tremendous wounds; but, at the top of a high and steep staircase, and at the aperture of an attic roof, the murderer had made himself a gallows. There was the ladder—the trap-door made a drop—the rope was adjusted—the noose was round the neck of the miserable man; before Stafford gaol or in Horsemonger-lane, he could scarcely have been executed with more formality or deliberation. His wife lying stark below, his home burning around him, he swung high up over the stairs.

* Respecting Mary Saxon's evidence, the *Manchester Guardian* states as follows:—"The first evidence of what had occurred seems to come from a woman named Mary Saxon, who, with her mother, occupies a front and a back cellar under the beer-house. Mrs. Saxon says that about three o'clock, while lying on a bed in her back cellar, she heard a heavy fall overhead, which startled her; that very soon afterwards she saw blood dropping through the ceiling; that she then said to her mother that 'he must have been killing her,' alluding to Robinson and his wife; but that her mother advised her not to do anything 'until the girls came home.' This Mrs. Saxon says she more readily consented to, as she had not heard any quarrelling. Mrs. Saxon further states, that about four o'clock she saw Robinson walking up and down Albert-street, in front of his house, and that he had then locked his front door, but had no hat on. She spoke to him, and asked, 'Where is Mrs. Robinson?' and on his answering, 'I don't know; I think she must have gone to our Eliza's,' Mrs. Saxon rejoined, 'No, she never has.' Strange as it may appear, Mrs. Saxon still gave no alarm, nor does she seem to have communicated any fear or suspicion to any person. Indeed, she says that she left Robinson in the street, 'with the tears rolling down his cheeks like a bewildered man.' Nothing more was known until the daughters returned home, about a quarter before seven o'clock. They were astonished at finding the house in darkness and the front door locked; and they were, of course, terrified at the tale which Mrs. Saxon told them. The aid of two neighbours, Thomas Kenny and Frank Waring, was obtained; a back door was forced, and the house was entered."

"Now this was the work of jealousy, of drunkenness, of moody and morbid irritability. Sensitive and passionate, the unhappy wretch, in the intervals of his amateur performances under palls or in a black scarf, would torture himself by fancies of his wife's infidelity; and frequently the expression of his face told her—although he never seems to have employed a threat—that he was debating within himself whether or not to become her murderer. At length the impulse of insanity overpowered this most miserable of men; and, first slaying his wife, then flying from himself into the streets, and not daring to answer a neighbour's question, he finally locked and barred himself in the house, set it on fire, prepared his own scaffold, and perished—to leave a scarecrow memory, and perhaps an effigy for some Midland Chamber of Horrors. What a climax to human life, in a civilized and Christian country!"

While the writer of this article rightly attributes the deeds committed by Robinson, and many of the more remarkable recent instances of murder, to insanity, and, moreover, properly inveighs against transcendental notions of irresponsibility, he is hampered with the more doubtful cases of disordered mind that occasionally come before our courts of law charged with murder—cases in which it is difficult to pronounce whether the mind is fettered morally alone, and is, consequently, responsible, or whether it is fettered physically, and is irresponsible. But the question with him, is one of expediency rather than right; yet, if example be required, incarceration would seem to offer as great a restrictive influence upon crime as the punishment of death, so far as we may judge from experience; and we presume that the expediency of hanging an irresponsible individual can scarcely be maintained.

This subject leads us almost directly to one—the hereditary transmission of moral peculiarities—which has been most ably discussed in a well-timed article published in the last number of the *British Quarterly Review*. The writer of this article examines very lucidly and learnedly the whole subject of *Physical and Moral Heritage*, and the views which he advances throw considerable light upon the moral characteristics of individuals like Robinson and Mary Saxon, as may be gathered from the following extract on the heritage of unsound mind, which is but one of several extracts that we should have liked to have laid before our readers had space permitted; but we commend the whole article to their attention:—

"The practical importance of this subject, in a popular point of view, consists in two facts—1. That there is a debateable ground of mental condition, which is not insanity in the eye of the law or of the physician, but which cannot possibly be spoken of as perfect mental soundness; and 2. That the various forms of slight and severe mental affection are naturally interchangeable and transformable by way of generation; thus, hysteria or chorea in one generation may become imbecility, mania, or epilepsy in the next or third. Insanity of any form in the parent may be represented in the offspring either by a similar affection, by sensory disorders (as deaf-dumbness, &c.), by epilepsy, by hysteria, or by the vague and undefined weaknesses or perversions of judgment, capacity, or will which we call unsoundness of mind.

"The general law with these neuroses is that, without special attention to the laws of hygiene, they increase in gravity and intensity from generation to generation; and thus young persons who weakly encourage hysterical habits, or the blind indulgence of impulses without the intervention of will and conscience, are laying the foundation for the most serious lesions of intellect or morals in after generations. For not only are the special vices of organization and function inherited in an aggravated form, but it is sad, yet certain, that there are individuals who, in their own person, inherit the sum of the perverted tendencies of many anterior generations. M. Morel, speaking of such beings, uses the following forcible expression:—

" 'A development sufficiently remarkable, of certain faculties, may give a different colour to the future of these unfortunate heritors of evil; but their intellectual existence is circumscribed within certain limits, which it cannot pass.

" 'The conditions of degeneration in which the heirs of certain faulty organic dispositions find themselves, are revealed not only by exterior typical characters easily to be recognised, such as a small, ill-formed head, predominance of a morbid temperament, spinal deformities and anomalies, &c., but also by the strangest and most incomprehensible aberrations in the exercise of the intellectual faculties and of the moral sentiments.'

"Our English law recognises as insane those who do not know right from wrong, and, considering their moral liberty as extinguished, views them as irresponsible. It recognises as sane those who do know right from wrong, and views them as responsible—as enjoying moral liberty: a very imperfect and faulty conception.

"Many of those who are called insane could tell in forcible language the difference between moral right and wrong, whilst many of those who mix daily in the affairs of men, and are considered sane, have no proper or practical conception of such differences. Now, if moral liberty means anything beyond a formula without interpretation, it means the power of choosing and acting according to the dictates of judgment, conscience, and will, in opposition to impulse and temptation. The impulse and the temptation being increased, and the faculties of judgment and will and the dictates of the conscience being both relatively and absolutely diminished, it follows necessarily that, in proportion to these changes, moral liberty is invaded, its powers curtailed, and responsibility to some extent modified. These are precisely the variations which we observe occurring in obedience to the law of heritage. All the qualities or lineaments of a parent are not equally inherited by the children, but divided amongst them. So in affections of the mind, it is not always the same and entire phase which is represented in the offspring, but this is analysed and the elements distributed. In one we have an impulsive nature, in which, between the idea and the act, there is scarcely an interval; in another, the proneness to yield to temptation of any kind—a feeble power of resistance, inherited either from the original or the acquired nature of the parent; in a third we have an imbecile judgment; in a fourth, an enfeebled vacillating will; in a fifth, or in all, a conscience by nature or habit torpid, and all but dormant. All these are the normal representatives of an unsound parentage, and all are potentially the parents of an unsound progeny; in all is moral liberty weakened; in all is responsibility not an absolute, but a relative idea.

"The man who inherits from his parents an impulsive or easily tempted nature, and an inert will and judgment, and commits a crime under the influence of strong emotion, can no more be placed in the same category of responsibility with a man of more favourable constitution and temperament, than can a man who steals a loaf under the pangs of starvation with the merchant who commits a forgery to afford him the means of prolonging a guilty career. We do not hesitate to say that these constitutional defects may be (and daily are) so combined as to produce almost complete irresponsibility, under a rational system of judgment, even in cases where the intellect, such as it is, remains coherent, and its possessor is accounted sane. Hence arises, in great measure, that strange insoluble problem of our race—the existence of what are called the 'DANGEROUS CLASSES;' a people who seem set apart to fill our gaols, our penitentiaries, our houses of correction, our penal settlements; a people at war with their kind—natural enemies of their brethren; a leaven leavening and infecting and drawing into the vortex of its own corruption even the comparatively sound elements of society; the pariahs of humanity, the despair of philanthropists, the opprobrium of legislation. It will not be by constantly repeated corrections that these classes will be reformed: 'Why should ye be stricken any more? Ye will revolt more and more;' but by a patient repetition of the means by which man as a race has been civilized. Successive generations, undergoing the process of elevation from barbarism, have been born not only into an improved and more favourable medium or condition of society, but also into an inheritance of faculties or aptitudes, intellectual and moral, refined and strengthened by the cultivation of those of their parents; and so it must be by successive attempts at the cultivation of the moral nature of these dangerous classes, that they, the barbarous elements of social life, must be redeemed from this present degraded condition, and enabled to transmit an improving and still improvable nature to their descendants."

In a subsequent paragraph of the same article we are told:—

"It is somewhat singular that, amongst a people so barbarous as the Chinese, we should find, in reference to these hereditary weaknesses and crimes, a custom worthy of, but not followed in, the most civilized nations. In examining a criminal, they do not only inquire into the facts of the crime itself, they examine most minutely into the temperament, complexion, and physical state of the accused; into the most trifling events of his former life; into everything that can throw any light upon motive or impulse; also, into the state of the parents and ancestors. Were this same rule systematically followed out in European courts of justice, we should very soon have a collection of the most valuable data for the solution of many hitherto insoluble problems, such as the general relations of organization to morality, of criminality to ignorance, education, insanity, and so forth. This excellent custom in the nation in question is accompanied, however, by a barbarity of punishment which we should by no means wish to see emulated. If a Chinese be convicted of lese-majesty, the law is, 'that he be cut into ten thousand pieces, and his sons and his grandsons be put to death.' It appears that a similar law exists in the code of Persia, but only as to the *letter*, never being acted upon."

In connexion with the subject of hereditary insanity, we may refer to an interesting meeting which was held in Edinburgh on the 3rd of February, for the purpose of forming a Society for the Education of Imbecile Children. The meeting was numerously attended, and Sir John J. Forbes occupied the chair. The following is a brief report of the principal speeches:—

"The Chairman expressed his gratification at the interest which was beginning, though somewhat tardily, to be felt in Scotland on a subject of so pressing and important a nature as the improvement of the physical, moral, and intellectual condition of this most unfortunate class of our fellow-creatures, which was, unhappily, much larger, he believed, than the public were at all aware of. After pointing out the distinction between the class of lunatics proper, and those more especially contemplated by the proposed society, he stated that, while the necessity of dealing with the former had long been recognised, the latter had either been confounded with them and subjected to confinement and restrictions which could only aggravate their unhappy condition, or, in the great majority of cases, they were left to wander through the country, most melancholy specimens of intellectual want, not only disagreeable but repulsive to the common sentiments of society. Sir John then adverted to some of the physical and moral causes to which imbecility was believed to be more or less directly attributable—specifying, among others, intemperance, insufficient and unwholesome or innutritious food, filthy and inadequate accommodation, and the squalor and misery thereby occasioned—and stated that, in almost all cases, idiocy was accompanied by defective muscular development. For a good many years, institutions devoted to the care and education of these unfortunates had existed in France, Switzerland, Austria, Prussia, Denmark, and, to a still greater extent, in the United States. Some time ago several were opened in England, but in Scotland the only attempts of the kind as yet had been made and carried on by private benevolence. The object of the present meeting was to endeavour to embody their sentiments in regard to this great question, and to express them in such an appeal to the benevolence to the country as might meet, not only with a wide recognition of its necessity on the part of individuals, but force on the conviction of their rulers the fact that this was an object paramountly demanding national interference. The numbers of the class in question had not yet been distinctly ascertained. Of course the proportion varied in different communities, and it still required a great deal of statistical inquiry before it could be accurately stated. It was believed, however, that there were about 3000 of the idiotic class in Scotland, and of these it was estimated that 600 were susceptible of improvement; but he was sorry to say that not one-tenth of these had yet an asylum where they might undergo a kindly course of treatment. The fact was well ascertained that this unfortunate deficiency in mental powers generally proceeded from an infirmity of bodily health, which tended to prevent the development of the physical, moral, and intellectual powers of the individual. The deficiency in the physical system naturally led to a distinct indication of their course of management. It was through the physical powers that they must approach the intellectual deficiency, and, by strengthening them, give confidence to the recipient of their treatment, and gradually enable the increased physical power to sustain and improve the intellectual development. This course had been carefully pursued in most of the foreign institutions, and it was a point which, he believed, the best medical authorities in this country were very anxious to place foremost in the scheme of treatment for these individuals; and there was also room for a rational hope that the gradual improvement of social arrangements among the poorer classes of the community, now happily, he believed, going forward, would tend to diminish the number and ameliorate the acerbity of the general class of cases. He then alluded to the admitted desirableness of a more distinct classification of cases than at present existed in lunatic asylums, and stated his conviction that, if such a classification had been carried out, a great number of lunatics, who had now become dangerous to society and themselves, and who required restraint, would never have arrived at that unhappy position. Sir John then stated that he had received communications from the Rev. Dr. Robertson, and from Dr. Poole of Aberdeen, expressing their sympathy with the object of the meeting, and their regret at being prevented from attending. He also read a letter from Lord Kinnaird, in which his lordship, after expressing his gratification on hearing of the present movement, says:—

"'My idea is, that it is impossible to meet the extent of the evil by private benevolence; but if receptacles for imbeciles on an extensive scale were established in country districts, they might be classified and maintained at a very *reasonable rate*, in which case both parents and parishes would gladly avail themselves of the benefit of such asylums. I think that a more correct investigation into the question of lunatics will show that a vast number of patients would be found better fitted for such establishments than for lunatic asylums; and, instead of counties being called upon to encounter the expense of providing lunatic asylums, less expensive buildings for harmless lunatics and imbeciles might be erected. It appears to me that the expense of erecting such buildings should be provided for by a general rate. Three establishments would, I think, be sufficient (at all events in the first instance) to meet the present requirements—a portion of the building being set apart for the training of children thus afflicted. If I meet with general support, I propose introducing, as you are aware, an amended Lunacy Act; and I should, in that case, make provision for two distinct classes of asylums, which would embrace, I presume, the object the meeting has in view.'

"Dr. Coldstream then addressed the meeting at some length, reviewing the history and present aspect of the movement on the Continent of Europe and in America. In this country, Dr. Abercrombie, in his *Thesis on Cretinism*, written in 1803, had suggested the adoption of means likely to ameliorate the condition of the fatuous. The subject was again, in 1819, brought before the public by Dr. Poole of Aberdeen; but, although forty years had elapsed since, no step had been taken in Scotland to carry the suggestions into practical effect. After referring to the establishment of a large asylum at Reigate in England, and to the institution erected at Baldovan, near Dundee, by Sir John and Lady Jane Ogilvy, Dr. Coldstream stated the circumstances under which the School and Home at Gay-field-square had been commenced in 1855. Though the experiment had not been made under such favourable circumstances as could have been desired, it had, on the whole, been a very successful, one, and demonstrated that, up to a certain point, the institution might be made nearly self-supporting—so much so, that the directors were solicitous to extend the benefits of the system by getting a society formed which would have more of a national than a local character. It was their desire to make the funds placed at their disposal go as far as possible, by eschewing costly buildings and other sources of lavish and often useless expenditure; and he indulged a very confident hope that their efforts in this important work would be seconded by the public; and, as an encouragement to those who took an interest in the work, he was authorized to say that a liberal friend had expressed his readiness to contribute 500*l.* to their funds immediately on the society being established. In the course of further observations, Dr. Coldstream said the directors wished the movement to be regarded as an educational one; they desired to make a fair trial of all the imbecile children—a considerable portion of whom were improvable—and they were extremely anxious that the young people should be kept as much as possible out of the category of lunatics, until every educational appliance had been exhausted on them in vain.

"Mr. W. H. Bell then read a statement as to the position of the provisional institution now in existence, from which it appeared that the establishment was now in its fourth year, that, from its commencement, 41 pupil-patients had been, for longer or shorter periods, in training, and that 24 were now within its walls. Besides these, there had been about 100 applications for admission, which the committee had been unable to entertain. The total receipts from the commencement of the undertaking had been 2463*l.* 18*s.* 11*d.*, and the expenditure, 2169*l.* 2*s.* 6*d.*—leaving a balance of 294*l.* 16*s.* 5d. in the treasurer's hands. The committee submitted that the present house in Gay-field-square was, in many respects, unfit for the purposes in view, and that, in order to secure the ultimate success of the project, it would be necessary that the public should come forward and put within their power the means of obtaining more suitable and commodious premises.

"Dr. Browne, one of the Commissioners in Lunacy, said that, while he wished to speak less as a member of a public board than as an individual who for a quarter of a century had been endeavouring—and endeavouring, he feared, unsuccessfully, chiefly for the reasons which had induced the promoters of the present movement to propose that it should take the form of an educational rather than a curative system—to train up the unhappy possessors of defective organizations in a public institution, he might state, that as a public officer he earnestly sympathized with the present movement. The matter had been brought under his observation in a manner and to an extent very different from that in which it had struck the public, and he even doubted if the unprofessional part of the present meeting were at all aware of the clamant nature of the case. He referred to the public sensation which followed the startling revelations made by a Parliamentary Blue-book some years ago as to the condition of the coal miners in this country, and stated his conviction that, if the full extent of the evil which this Society proposed to attempt to alleviate were made known, public feeling would be aroused in a still greater degree. One of the chief objects of the Society, therefore, he thought, should be to place the information already collected before the public, and to obtain additional statistics on the subject. The statistics of lunacy were still very defective, but enough was known to render it certain, that in any county numbering 150,000 inhabitants, there would be found 600 persons afflicted with insanity in one form or another, and out of these it was safe to conclude that about 200 came under the generic name of idiots. He was glad to hear that the directors of the new Society proposed to be very modest in their buildings, and he would also recommend them to be modest in their announcements of the results expected by them, because it was the fact that, on the Continent, much disappointment had been caused, not by the failure of the system followed to effect a great deal of practical good, but, by its failure to effect all the good which had been prophesied by its too sanguine friends. The directors must not expect, in the great majority of cases, to raise up the imbeciles placed under their care to the majesty of perfect men, but still there remained a large margin for their efforts. There were three things which experience had led him to believe they could effect. In the first place, they would be able to educate and train up to some considerable degree of intellectual capacity at least a fifth or a sixth of the whole number; and, by doing so, they would, in the second place, greatly increase the happiness of these beings; for whatever might be the popular notion, it was a fact well known to professional men that they existed in a state of intense misery and wretchedness. Thirdly, it would be possible to bring them to such a point of muscular and intellectual development that they would be enabled to contribute to their own support, and, in some cases, to support themselves altogether. He concluded by assuring the directors of the new society that they had the best wishes of himself and his colleagues in the very important task which they had set before them.

"Dr. Mitchell, Assistant Lunacy Commissioner, briefly expressed the great interest which he had long taken in the object of the present movement, and his cordial wishes for its success.

"A vote of thanks having, on the motion of Dr. M'Lagan, been passed to Sir J. Forbes for presiding, the meeting separated."

The police records of the quarter contain a curious case of a woman throwing her child out of the window under the influence of a dream. The following report of the case appeared in the *Express*, of January 5:—

"Yesterday the Marylebone Police-court was crowded to excess in consequence of a report which had been circulated that a woman was in custody for killing her child by throwing it from a first-floor window into the street. The rumour with regard to the murder happily turned out to be untrue; but it will be seen from the subjoined evidence that it was a providential circumstance that the lives of three children were not sacrificed by their mother while acting under the alleged influence of a dream.

At two o'clock the prisoner, Esther Griggs, was placed at the bar before Mr. Broughton.

"Mr. Lewis, of Ely-place, appeared for her; and Mr. Tubbs, relieving overseer of Marylebone, attended on the part of the board of guardians of the parish to watch the case.

"The prisoner, who evidently felt the serious situation in which she was placed, was seated during the proceedings.

"The first witness called was

Sergeant Simmonds, 20 D, who said—At half-past one o'clock this morning, while on duty in East-street, Manchester-square, I heard a female voice exclaim, 'Oh, my children; save my children!' I went to the house No. 71, from whence the cries proceeded, and the landlord opened the door. I went upstairs, accompanied by two other constables, and while making our way to the first-floor front room, I heard the smashing of glass. I knocked at the door, which I found was fastened, and said, 'Open it, the police are here.' The prisoner, who was in her night-dress, kept on exclaiming, 'Save my children;' and at length, after stumbling over something, let me and my brother officers in. When we entered we found the room in total darkness, and it was only by the aid of our lanterns that we could distinguish anything in the room. On the bed there was a child, five years old, and another, three years of age, by her side. Everything in the place was in great confusion. She kept crying out, 'Where's my baby? Have they caught it? I must have thrown it out of window.' The baby must have been thrown out as I was going upstairs; for, before getting into the room I heard something fall. I left a constable in charge of the prisoner, and I ascertained that the child, which had been thrown from the window, had been taken to the infirmary of Marylebone workhouse. She told me that she had been dreaming that her little boy had said that the house was on fire, and that what she had done was with the view of preventing her children from being burnt to death. I have no doubt (added witness) that if I and the other constable had not gone to the room, all three of the children would have been flung out into the street.

"Mr. Broughton.—How long do you suppose the cry of 'Oh, save my children!' continued?

"Witness.—I should think nearly five minutes. (In continuation, he said he went to 38, Harley-street, where the husband lives in the service of a gentleman, and gave him information of what had occurred. The injured infant was only 18 months old.)

"By Mr. Lewis.—From the excited state in which the prisoner was, I did not at the time take her into custody. She went to the infirmary along with her husband to see how the child was going on, and what hurt it had sustained. I had understood that the surgeon had said it was a species of nightmare which the prisoner was labouring under when the act was committed. The window had not been thrown up. The child was thrust through a pane of glass, the fragments of which fell into the street.

"Humphreys, 180 D.—I heard the breaking of glass, and saw what I imagined to be a bundle come out of the window, and on taking it up I found it to be a female infant. There was blood running from its temples, and it was insensible, I took it to the infirmary.

"Pollard, 314 D.—I heard loud cries of 'Oh, save my children!' and when I was in her room she said, 'Has anybody caught my baby, Lizzy?' One of the little boys, three years old, and who was clinging to his mother, had blood upon his clothes. He had upon his breast some marks which appeared to have been caused by cuts from glass. The sergeant left me to take care of the prisoner while he went for her husband. She told me she had no wish to hurt any of her children, and that it was all through a dream.

"Mr. Henry Tyrrwhitt Smith, surgeon at the Marylebone infirmary, was next called, and said that when the infant was brought to him, soon after one in the morning, he found upon examining it that it was suffering from concussion of the brain. It was quite insensible and decidedly in danger now.

The parietal bone is broken, and death might ensue in the event of an effusion of blood on the brain.

"By Mr. Lewis.—I cannot say that I have not heard of an instance where parties have committed acts to which a dream had impelled them.

"Mr. Lewis submitted to the magistrate that there had been no attempt to murder the infant. The prisoner had always evinced a kindly feeling towards her children, and he (the learned gentleman) hoped that the magistrate would allow the husband to have her under his care during the temporary remand which of course would take place. The dream under which the act was committed showed that she had not at the time any consciousness of what she was doing.

"Mr. Tubbs said he did not attend in the capacity of a prosecutor, but he appeared on behalf of the board of guardians, and he put it to the magistrate whether there would be any objections, under the circumstances, to allow the prisoner to be bailed, her husband being security for her reappearance.

"Mr. Broughton *considered that it would be a most dangerous doctrine to lay down to say, that because a person was dreaming whilst committing an offence that they were not culpable for their acts* (!). A woman, on these grounds, might get up in the middle of the night and cut her husband's throat, and when brought up for the offence turn round and say that she had done the act whilst under the influence of a dream. He (the worthy magistrate) considered the case to be one of a serious nature, and in the event of death ensuing an inquest would be held on the body. He could not think of taking bail in so serious a case, but would remand the prisoner till Tuesday next, and during her present excited state she would be taken care of in the infirmary.

"The prisoner was then removed to the cells by Ansted, the gaoler, sobbing most bitterly."

The Recorder, at the subsequent sessions at the Central Criminal Court, in his address to the grand jury, took a somewhat more rational view of the case than that entertained by Mr. Broughton. "If the prisoner," said the Recorder, "really did the act under the idea that it was the best mode of insuring the safety of the child, it appeared to him, that under such circumstances, it would be a question whether the grand jury would be justified in coming to the conclusion that the prisoner was guilty of a criminal act." The grand jury threw out the bill.

The judgment of Lord Chief Justice Cockburn upon the case of Robinson *v.* Robinson and Lane, in the Court for Divorce and Matrimonial Causes (March 2), merits quotation in part. The particulars of the case have been fully made known from time to time in the daily papers and need not be recapitulated. His lordship said:—

"This was a suit for a divorce *à vinculo*, on the ground of adultery. The case was peculiar and remarkable in its character and circumstances. The only evidence to support the case of the husband, the petitioner, consisted of certain alleged admissions of the wife, the respondent, without any corroborative evidence, direct or indirect, to support them. . . . . On the part of Mrs. Robinson it was strongly contended that these narratives were the insane delusions of a diseased mind. To sustain that proposition, evidence was adduced that the respondent had for many years been labouring under disease, and the Court was assured on the highest medical authority that the effect of such disease was sometimes to produce mental derangement of the most painful nature with reference to sexual feelings, and an insane belief of having committed unchaste acts which had no existence except in the mind of the woman confessing them. *The Court found, however, nothing in*

*this case which would warrant it in concluding that the scenes narrated by the respondent were the delusions of a disordered mind. Had they been so, the Court would have no doubt found, what was usual in such cases, the statement or confession of them to others*, not a mere recording of them, among the other events of her life, in a secret journal to be seen by no eye but her own. *In cases of mania and delusions of this character, self-accusation seemed always to be a feature of the disease.* Probably, too, the Court would have found more distinct and unequivocal statements of the full consummation of her desires than were to be met with in the diary. Certainly they would not have found in so many instances complaints of imperfect pleasure or of painful disappointment. As the Court could not, therefore, adopt the view suggested as to the insane unreality of the narrative of Mrs. Robinson, it became necessary to consider the effect of this remarkable document, and to see whether they could conclusively collect from it that adultery had in fact taken place. . . . . . *In no instance could they find a clear and unequivocal admission of adultery having taken place.* The strongest passage was that in which the respondent stated, that after one of these interviews Dr. Lane desired her to take care to 'obviate consequences;' but even there the 'consequences' referred to might have been those of discovery or detection, and not those resulting from an adulterous connexion. It was true that where they were dealing with admissions by a wife of criminal and indecent familiarities, bordering upon and, morally speaking, partaking of adultery, the disposition of the Court would, for obvious reasons, be to give the fullest effect to the language of those admissions. But in this case there were considerations which led the Court to think that the language of Mrs. Robinson in her diary must be construed by a different rule. The Court was dealing with one whom an ardent imagination and a passionate nature too often led away beyond the bounds of reason and truth, and who, in all relating to her intercourse with men for whom she had conceived a partiality, was prone to exaggerate and over-colour every circumstance which tended to her gratification. There was a striking instance of this in her interview with Mr. Thom, where she magnified into the most serious importance circumstances which were proved to have been of the most ordinary and trivial character. In the present instance this tendency would be more strongly brought into play by the strength and ardour of her passion for Dr. Lane. It was plain that she dwelt with impure gratification on the portraiture of these scenes, and on the details of the guilty endearments and caresses which she narrated. *It was impossible to say how much of all this might not be the work of an imagination corrupted by sensuality and dwelling with morbid satisfaction on its own impure creations, or how far any groundwork of fact might be distorted or overcharged by the fanciful additions of the writer. The Court was, at all events, of opinion that all that came from such a quarter on such a subject, far from being taken as a ground for drawing further inferences of criminality, must be received with very great allowance and distrust.* Having no other evidence than the statements of a writer in whose judgment and fidelity to truth in the particular matter the Court could place no reliance, and whom it believed capable of distorting and discolouring facts to gratify her disordered fancy and morbid passion, it would have had great doubt whether, if the admissions had amounted to a clear acknowledgment of adultery, it could have given effect to them; but, looking to the ambiguous character of the expressions, coupled with the reiterated complaints of the writer of the absence of equal ardour on the part of Dr. Lane, it could not, under all the circumstances of the case, come to the conclusion that there was any admission of adultery upon which it would be justified in acting. It was true that from evidence of acts of guilty familiarity the Court or a jury would have no hesitation in inferring actual adultery, but they were here dealing with confessions made by a party who seemed to have every disposition to overstate rather than to suppress, and who, in these admissions, must be taken to have gone to the utmost limits of reality. To statements so made it was not open to the Court to add anything by way of inference. It was unnecessary to determine whether the whole of Mrs. Robinson's revelations were imaginary, or how much was to be set down as fiction and how much as fact; it was enough that, on the whole, the Court came to the conclusion that it had no evidence of adultery before it on which it would be justified in pronouncing a sentence of divorce. It regretted the position of Mr. Robinson, who remained burdened with a wife who had placed on record the confession of her misconduct, or, at all events, even taking the most favourable view, of unfaithful thoughts and unchaste desires; but redress could only be afforded by that Court on legal proof of adultery, and that proof the Court *could not find in the incoherent statements of a narrative so irrational and untrustworthy as that of the respondent.* Entertaining this opinion of the evidence derived from the journal, it would be unnecessary to enter into the consideration of the evidence given by Dr. Lane, and therefore the only course open to the Court was to dismiss the petition."

The facts of main importance legally in this case were the entire absence of corroborative evidence, and the insufficiency of the documents produced to support the charges ought to be established by them. The facts of main importance medically were the existence of uterine disease in the respondent, and the extravagance and incoherency of the statements contained in her diary. How incoherent and untrustworthy those statements were is sufficiently set forth in the last sentence but one of the judgment. Why an incoherent statement should be sufficient to exculpate an individual legally, and yet at the same time be coherent enough to condemn the same person morally, as laid down by the Lord Chief Justice, is more than we can say. Neither can we explain by what process his lordship can satisfy himself respecting the soundness of the medical dicta he has laid down, and yet reject the highest medical testimony. It is needless to dwell upon the utter falsity of the notions, that self-accusation or confessions to others are essential characters of the disease the respondent suffered from; and it is to be feared that the decision in this case, in so far as the question of morality is concerned, may, by diverting attention from the medical aspect in which Mrs. Robinson's mental condition ought to be viewed, have not only a most unhappy effect in her case, but also in other cases of a similar character which, unfortunately, exist, but which hitherto have been rightly appreciated and understood by friends and relatives.

We shall defer any notice of the proposed alterations both in the English and Irish Lunacy Laws until a subsequent period.

Before closing our retrospect, it is necessary that we should notice an event of no small interest to the psychologist, the publication of Sir William Hamilton's Lectures, embracing his metaphysical and logical courses, (*Wm. Blackwood and Sons.*) The work is edited by the Rev. R. L. Mansel, B.D., the Reader in Moral and Metaphysical Philosophy at Magdalen College, Oxford, and well-known Bampton Lecturer of 1858, and John Veitch, M.A., who was assistant to Sir Wm. Hamilton, and read his lectures to the class during his last illness. The work will consist of four volumes, of which the two first, containing the metaphysical course, are already out of the press. We propose to notice these volumes at large in our next number, but in the meantime we may remark, that their rare interest can only be rightly appreciated by a personal examination.

